# 2,4,6-Trihydroxy-3-geranyl acetophenone suppresses vascular leakage and leukocyte infiltration in lipopolysaccharide-induced endotoxemic mice

**DOI:** 10.1080/13880209.2021.1933083

**Published:** 2021-06-22

**Authors:** Yee Han Chan, Nazmi Firdaus Musa, Yi Joong Chong, Siti Arfah Saat, Faizul Hafiz, Khozirah Shaari, Daud Ahmad Israf, Chau Ling Tham

**Affiliations:** a Department of Biomedical Science, Faculty of Medicine and Health Sciences, Universiti Putra Malaysia, Serdang, Malaysia; b Laboratory of Natural Products, Institute of Bioscience, Universiti Putra Malaysia, Serdang, Malaysia; c Department of Chemistry, Faculty of Science, Universiti Putra Malaysia, Serdang, Malaysia

**Keywords:** tHGA, LPS, endothelial hyperpermeability, vascular inflammation

## Abstract

**Context:**

Lipopolysaccharide (LPS) exacerbates systemic inflammatory responses and causes excessive fluid leakage. 2,4,6-Trihydroxy-3-geranyl acetophenone (tHGA) has been revealed to protect against LPS-induced vascular inflammation and endothelial hyperpermeability *in vitro.*

**Objective:**

This study assesses the *in vivo* protective effects of tHGA against LPS-induced systemic inflammation and vascular permeability in endotoxemic mice.

**Materials and methods:**

BALB/c mice were intraperitoneally pre-treated with tHGA for 1 h, followed by 6 h of LPS induction. Evans blue permeability assay and leukocyte transmigration assay were performed in mice (*n* = 6) pre-treated with 2, 20 and 100 mg/kg tHGA. The effects of tHGA (20, 40 and 80 mg/kg) on LPS-induced serum TNF-α secretion, lung dysfunction and lethality were assessed using ELISA (*n* = 6), histopathological analysis (*n* = 6) and survivability assay (*n* = 10), respectively. Saline and dexamethasone were used as the negative control and drug control, respectively.

**Results:**

tHGA significantly inhibited vascular permeability at 2, 20 and 100 mg/kg with percentage of inhibition of 48%, 85% and 86%, respectively, in comparison to the LPS control group (IC_50_=3.964 mg/kg). Leukocyte infiltration was suppressed at 20 and 100 mg/kg doses with percentage of inhibition of 73% and 81%, respectively (IC_50_=17.56 mg/kg). However, all tHGA doses (20, 40 and 80 mg/kg) failed to prevent endotoxemic mice from lethality because tHGA could not suppress TNF-α overproduction and organ dysfunction.

**Discussion and conclusions:**

tHGA may be developed as a potential therapeutic agent for diseases related to uncontrolled vascular leakage by combining with other anti-inflammatory agents.

## Introduction

Inflammation is a localized protective response elicited by tissue injury or destruction that serves to destroy or sequester the injurious agent and tissue (Fleischmann et al. 2016). Sepsis, an infection-induced systemic inflammatory response, is one of the most common causes of death worldwide (Fleischmann et al. 2016). Despite significant advances in the development of antimicrobial chemotherapy and anti-infectious therapy, sepsis remains a significant cause of morbidity and mortality in humans (Cohen et al. 2015). The prominent induction of sepsis is largely caused by the dysregulation of vascular permeability and systemic inflammatory responses, characterized by the excessive accumulation of proinflammatory mediators. In the manifestation of sepsis, the host’s pathogen recognition receptors recognize and then bind in pathogen-associated molecular patterns (PAMPs), leading to the impaired vascular integrity, and subsequently the development of progressive subcutaneous and body cavity oedema. As tissue oedema is not benign, the accumulation of interstitial fluid could hinder organ function by increasing the distance required for oxygen diffusion. As blood vessels are lined with endothelial cells, vascular leakage and tissue oedema are the main causes of endothelial dysfunction in sepsis (Chen 2017). Remarkably, pathogen attacks could also provoke a storm of various proinflammatory cytokines, including tumour necrosis factor-alpha (TNF-α), interleukin-6 (IL-6) and interferon-gamma (IFN-γ) (Hartati et al. 2017). The increased proinflammatory cytokines will then mediate the expression of inducible nitric oxide synthase (iNOS) by immune cells to catalyse the over-production of nitric oxide in the presence of immunogenic or inflammatory stimuli (Soufli et al. 2016). This overwhelming inflammatory response activates an innate immune response, along with cytokine secretion and neutrophil accumulation (Hartati et al. 2017). Collectively, the vascular dysfunction and the exacerbation of inflammatory responses result in interstitial fluid leakage and a compromised immune system, leading to organ failure, septic shock and death (Crouser and Matthay 2017).

Endotoxemia is a condition in which an endotoxin, primarily lipopolysaccharide (LPS), is present in the bloodstream due to bacterial infection (bacteraemia). This pathological event is known as 'blood poisoning,' which is deleterious to the host, as the endotoxin can be carried throughout the body and provoke hyperinflammatory responses that will eventually lead to sepsis (van Poelgeest et al. 2018). LPS is an outer membrane component of Gram-negative bacteria that binds to a Toll-like receptor 4 (TLR4) to trigger systemic inflammation and sepsis progression (Li et al. 2017). When LPS is engaged locally or systemically in high concentrations, it activates TLR4 and triggers a series of signalling pathways, which then leads to impaired vascular integrity and endothelial hyperpermeability as a result of a compromised junctional complex. Notably, once an inflammatory response is initiated, the activated signalling pathways will provoke the upregulation of various proinflammatory mediators, such as nuclear factor-kappa B (NF-κB), mitogen-activated protein kinases (MAPKs), and cytokines and chemokines, further exacerbating vascular leakage in an unrestricted manner (Li et al. 2017). These pathological conditions will lead to multiple organ failure and septic shock, eventually causing death (Polat et al. 2017).

Currently, the intravenous injection of antibiotics, intravenous fluid and vasopressor agents, such as norepinephrine and dopamine, are widely employed to clinically treat sepsis. For inflammatory diseases, various drugs, such as glucocorticoids and immune modulators, are prescribed to patients (Heming et al. 2018). However, these clinically prescribed drugs cause numerous, severe side effects, including liver damage, osteoporosis and diabetes (Oray et al. 2016). Nonsteroidal anti-inflammatory drugs (NSAIDs) and herbal compounds not only have therapeutic values in the treatment of disorders related to endothelial hyperpermeability and vascular inflammation, but also pose little or no side effects to patients (Pereira-Leite et al. 2017). Therefore, this situation has prompted researchers to explore novel approaches using natural products as a potential therapy for treating vascular inflammatory disorders, such as endotoxemia and sepsis.

2,4,6-Trihydroxy-3-geranyl acetophenone (tHGA) is a bioactive phloroglucinol compound that was originally isolated from the young leaves of a medicinal plant called *Melicope ptelefolia* Champ Ex. Benth (Rutaceae) (Shaari et al. 2011). Phloroglucinol is a class of compounds that have proven abilities to attenuate the LPS-mediated barrier disruption in human endothelial cells (Bae et al. 2012; Chong et al. 2016). Therefore, intensive studies on the molecular mechanism of tHGA are propitious to the clinical therapy of vascular inflammatory diseases. Previous studies by the current research group have demonstrated the ability of tHGA to exhibit barrier protective properties *in vitro*, mainly via the inhibition of vascular inflammation and endothelial hyperpermeability (Chong et al. 2016). Specifically, the *in vitro* anti-inflammatory effect of tHGA is attributed to its ability to reduce monocyte adhesion and transmigration across the endothelial monolayer via the inhibition of cell adhesion molecule (CAM) expression and prostaglandin production (Chong et al. 2016). As an *in vivo* study is more representative and reliable than an *in vitro* one, this study determined the *in vivo* protective effects of tHGA against endotoxemia, which is a condition exaggerated by LPS-mediated endothelial permeability. Specifically, this study investigated the effects of tHGA on vascular leakage, as well as the leukocyte infiltration across the vascular barrier, the secretion level of proinflammatory TNF-α, the histopathological changes in the lungs, and the survivability of LPS-induced endotoxemic mice.

## Materials and methods

### Reagents

Phosphate buffer saline (PBS), LPS derived from *Escherichia coli* O111:B4, Tween-80, Tween-20, sodium chloride (NaCl), potassium chloride (KCl), sodium phosphate dibasic (Na_2_HPO_4_), monopotassium phosphate (KH_2_PO_4_), 3,3′,5,5′-tetramethylbenzidine (TMB) and citric acid were purchased from Sigma-Aldrich (St. Louis, MO). Dimethyl sulphoxide (DMSO) and dexamethasone were purchased from Merck Millipore (Darmstadt, Germany). Absolute ethanol, 95% ethanol and 4% paraformaldehyde were purchased from HmbG Chemical (Hamburg, Germany). Bovine serum albumin (BSA), enzyme-linked immunosorbent assay (ELISA) DuoSet kit for mouse TNF-α, xylazine and ketamine, xylene, paraffin wax and dibutylphthalate polystyrene xylene (DPX) mounting medium were purchased from Amresco (Solon, OH), R&D System (Minneapolis, MN), Troy Laboratories Pty. Ltd. (Glendenning, Australia), Bendosen (Shah Alam, Malaysia), Leica Biosystems (Nußloch, Germany) and Essen-Haus Sdn. Bhd. (Subang Jaya, Malaysia), respectively.

### Synthesis of 2,4,6-trihydroxy-3-geranyl acetophenone

tHGA was synthesized according to previously described methods (Ismail et al. 2012). First, phloroacetophenone (1.000 g, 6 mmol), geranylbromide (0.876 g, 4.80 mmol) and anhydrous potassium carbonate (0.415 g, 3.00 mmol) were mixed well in dry acetone (3.5 mL) and then refluxed for 6 h. Then, the reaction mixture was filtered and evaporated under reduced pressure to give an oily orange residue. This residue was later purified via flash column chromatography on Sigel (petroleum ether:EtOAc, 10:1) to produce tHGA in the form of a light yellow powder; mp 128–130 °C. ^1^H NMR (CD_3_OD), *δ*_H_ 1.58 (3H, s, Me), 1.63 (3H, s, Me), 1.76 (3H, s, Me), 2.64 (3H, s, COMe), 1.96 (2H, q, *J* = 7.5 Hz), 2.06 (2H, m), 3.21 (2H, d, *J* = 6.5 Hz), 5.08 (1H, t, *J* = 7 Hz), 5.20 (1H, t, *J* = 6.5 Hz), 5.92 (1H, s, ArH); IR (KBr) *ν*_max_ 3405 and 1627 cm^–1^; EIMS *m*/*z* (%) [M] + 304 (38), 289 (3), 261 (9), 235 (25), 181 (100). The purity of tHGA was noted as more than 99%.

### Experimental animals

A total of 132 healthy male BALB/c mice (5 weeks old) were purchased. Then, 36 mice were used for Evans blue permeability assay and the leukocyte transmigration assay. Another 36 mice were used for ELISA and the histopathological analysis. The remaining 60 mice were used for the survivability assay. The mice were acclimatized for seven days in cages with a surrounding temperature of 24 ± 1 °C, relative humidity of 70–80% and a 12 h light/dark cycle for seven days prior to the experiments. The mice were fed with standard laboratory chow and water *ad libitum*. Six experimental groups were designated for all assays, with each group of mice randomly allocated into individual cages.

For Evans blue permeability assay and the leukocyte transmigration assay (*n* = 6), the mice were divided into six experimental groups as follows:

1. Normal group: no treatment, induced with PBS intraperitoneally (i.p.).
2. LPS control group: no treatment, induced with 15 mg/kg LPS (i.p.).
3. Drug control group: pre-treated with 3 mg/kg dexamethasone (i.p.), induced with 15 mg/kg LPS (i.p.).
4. tHGA-pre-treated LPS-induced group 1: pre-treated with 2 mg/kg tHGA (i.p.), induced with 15 mg/kg LPS (i.p.).
5. tHGA-pre-treated LPS-induced group 2: pre-treated with 20 mg/kg tHGA (i.p.), induced with 15 mg/kg LPS (i.p.).
6. tHGA-pre-treated LPS-induced group 3: pre-treated with 100 mg/kg tHGA (i.p.), induced with 15 mg/kg LPS (i.p.).

For other assays, the mice were divided into six experimental groups (*n* = 6 for ELISA and histopathological analysis; *n* = 10 for survivability assay), as follows:

1. Normal group: no treatment, induced with PBS (i.p.).
2. LPS control group: no treatment, induced with 15 mg/kg LPS (i.p.).
3. Drug control group: pre-treated with 3 mg/kg dexamethasone (i.p.), induced with 15 mg/kg LPS (i.p.).
4. tHGA-pre-treated LPS-induced group 1: pre-treated with 20 mg/kg tHGA (i.p.), induced with 15 mg/kg LPS (i.p.).
5. tHGA-pre-treated LPS-induced group 2: pre-treated with 40 mg/kg tHGA (i.p.), induced with 15 mg/kg LPS (i.p.).
6. tHGA-pre-treated LPS-induced group 3: pre-treated with 80 mg/kg tHGA (i.p.), induced with 15 mg/kg LPS (i.p.).

All animal experiments were approved by Institutional Animal Care and Use Committee (IACUC) under Universiti Putra Malaysia and performed in accordance to the approved guidelines (UPM/IACUC/AUP-R019/2016).

### Evans blue permeability assay

The permeability assay was performed based on previously described protocols with some modifications (Bae et al. 2012). A total number of 36 mice were used in this assay, where the mice were randomly allocated into six experimental groups (*n* = 6) as described in ‘Experimental animals’ section. Mice were pre-treated with PBS, tHGA (2, 20 and 100 mg/kg) or dexamethasone (3 mg/kg) via i.p. route for 1 h, followed by the induction of LPS (15 mg/kg) via i.p. route for 6 h. Evans blue (1%) was then injected intravenously (i.v.) and allowed to circulate in the blood vessels for 30 min. Next, the mice were sacrificed, and 2 mL of ice-cold PBS was injected into the mice peritoneal region to obtain a peritoneal wash. Permeated Evans blue was measured spectrophotometrically using a microplate reader (UVM 340, ASYS Hitech GmbH, Eugendorf, Austria) at a wavelength of 620 nm. The plate correction factor was fixed at 740 nm. The data were expressed in µg per mouse based on a standard curve.

### Leukocyte transmigration assay

The leukocyte transmigration assay was performed based on previously described protocols (Hudalla et al. 2018). This assay used the same mice from Evans blue permeability assay by collecting peritoneal wash samples from the mice. The peritoneal wash samples collected were then counted using a haemocytometer upon staining with Turk’s solution.

### Enzyme-linked immunosorbent assay (ELISA)

A total number of 36 mice were used in this assay, where the mice were randomly allocated into six experimental groups (*n* = 6) as described in ‘Experimental animals’ section. After seven days of acclimatization, BALB/c mice were pre-treated with PBS, tHGA (20, 40 and 80 mg/kg) or dexamethasone (3 mg/kg) (i.p.) for 1 h, followed by 6 h of LPS induction (i.p.) according to the experimental design. Six hours later, the mice were anaesthetized with xylazine and ketamine. About 0.6 mL of blood was then collected via cardiac puncture, followed by centrifugation to obtain the blood serum. The serum was used to measure the level of proinflammatory cytokine TNF-α, using the ELISA kit, according to the manufacturer's instructions (R&D System, Minneapolis, MN, catalogue no.: DY410). The results were then obtained using a microplate reader at 450 nm (Tecan Austria GmbH, Grödig, Austria) and expressed in pg/mL.

### Histopathological examination of lungs

Histopathological analysis used the same mice from ELISA assay by collecting lung samples from the mice. The lung samples collected were excised and fixed with 10% formalin. Lung tissues were then fixed in 4% buffered paraformaldehyde, dehydrated in a series of graded ethanol and embedded in paraffin. After that, sections of tissue were cut into 5 µm size on a rotary microtome and fished onto glass microscopic slides. The sections were then cleared, hydrated and stained with haematoxylin and eosin (H&E) for the histopathological evaluation of inflammatory cell infiltration and alveolar wall thickness, according to standard protocols. The slides were coded to prevent observer bias during evaluation. All tissue sections were then visualized using a light microscope (Leica DM2500, Allendale, NJ) and photographed with a digital camera.

### Survivability assay

The survivability assay was performed based on previously described protocols (Su et al. 2017). A total number of 60 mice were used in this assay, where the mice were randomly allocated into six experimental groups (*n* = 10) as described in ‘Experimental animals’ section. The mice were pre-treated with PBS, tHGA (20, 40 and 80 mg/kg) or dexamethasone (3 mg/kg) via i.p. route for 1 h, followed by LPS (15 mg/kg) via single i.p. injection. The mice were then returned to their individual cages and monitored for survivability at intervals of 6 h up to 168 h. Dead mice were removed to prevent contamination.

### Statistical analysis

All experiments described were performed three times. The results were expressed as mean ± S.E.M. Statistical analyses were performed using the Statistical Package for the Social Sciences (SPSS) 21.0 (SPSS Inc., Chicago, IL). A Kaplan–Meier log-rank test was employed to analyse the results of survivability assay. One-way analysis of variance (ANOVA) was conducted, followed by post hoc Tukey’s honest significant difference test to compare the result between different experimental groups. In this case, *p* ≤ 0.05 was considered statistically significant. Percentage of inhibition and IC_50_ were calculated by using GraphPad Prism 8 (La Jolla, CA).

## Results

### Effect of tHGA on vascular leakage in LPS-induced BALB/c mice

**Figure 1.**
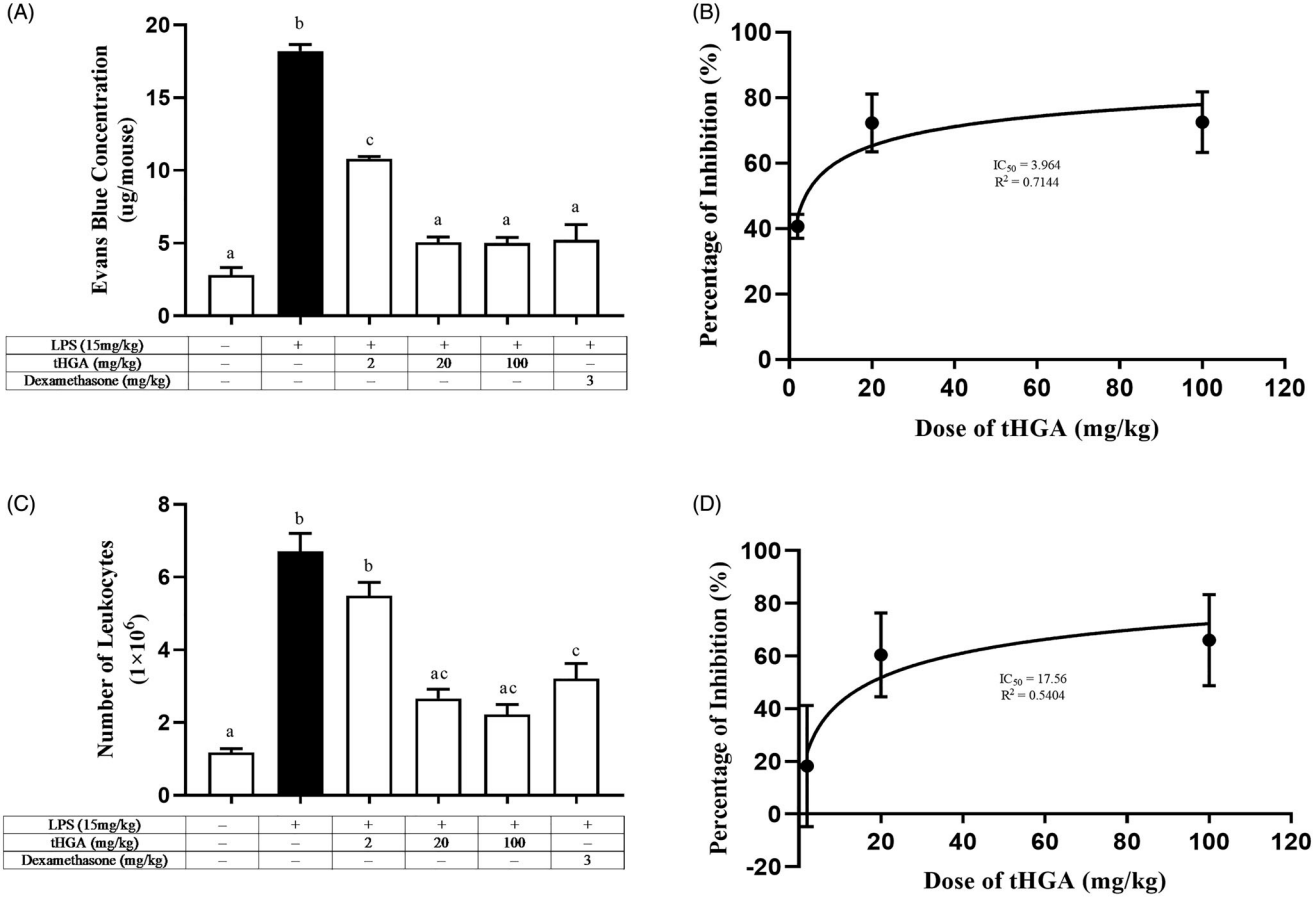
Effect of tHGA on (A) vascular leakage with (B) its IC_50_ and (C) leukocyte infiltration with (D) its IC_50_ in LPS-induced BALB/c mice. Mice were pre-treated with different doses of tHGA or dexamethasone prior to LPS induction. After 6 h, Evans blue was injected and peritoneal fluid was obtained 30 min later to determine the total vascular leakage. For leukocyte transmigration assay, peritoneal fluid was removed, centrifuged and the cell pellet was stained with Turk’s solution before being counted by using haemocytometer. Data are expressed in mean ± S.E.M. (*n* = 6), with groups that have no superscript letter in common are significantly different from each other (*p* ≤ 0.05).

Evans blue permeability assay was performed to examine the effect of tHGA on LPS-induced vascular leakage in BALB/c mice. The mice were pre-treated with tHGA in doses of 2, 20 and 100 mg/kg for 1 h, followed by 6 h of LPS induction to induce endotoxemia. As shown in Figure 1(A), the amount of permeated Evans blue in the LPS control group increased by 6.7-fold as compared to the normal group. However, upon tHGA pre-treatment, the mice showed significantly reduced amounts of permeated Evans blue at all tested doses of tHGA. In comparison to the LPS control group, tHGA inhibited the leakage of Evans blue by 48%, 85% and 86% at 2, 20 and 100 mg/kg, respectively (Figure 1(A)), with the IC_50_ value of 3.964 mg/kg (Figure 1(B)). For dexamethasone, which served as the drug control, 84% inhibition was observed. These results suggest that tHGA successfully attenuated LPS-induced vascular leakage. Interestingly, it is also found that the inhibitory effect was not dose-dependent as there was no significant difference between 20 and 100 mg/kg tHGA in reducing LPS-induced vascular leakage, indicating that tHGA might exert its optimum inhibitory effects at doses ≤20 mg/kg.

### Effect of tHGA on leukocyte infiltration in LPS-induced BALB/c mice

A leukocyte transmigration assay was performed to determine the number of leukocytes capable of crossing the intercellular gap during the barrier disruption in LPS-induced mice. The mice were pre-treated with increasing doses of tHGA, which are 2, 20 and 100 mg/kg for 1 h, before 6 h of LPS induction. In comparison to the normal group, upon LPS induction, the total transmigrated leukocytes increased by 5.7-fold (Figure 1(C)). However, in comparison to the LPS control group, 20 and 100 mg/kg tHGA reduced the number of migrated leukocytes in the peritoneal cavity by 73% and 81%, respectively, with the IC_50_ value of 17.56 mg/kg (Figure 1(D)). The drug control produced a weaker reducing effect of 63%. In line with the results from LPS-induced vascular leakage, tHGA at the doses of 20 and 100 mg/kg significantly suppressed LPS-induced leukocyte infiltration. Similarly, there was no significant difference between 20 and 100 mg/kg in terms of their inhibitory effects on LPS-induced leukocyte infiltration, once again suggesting that tHGA might exert its optimum inhibitory effects at doses ≤20 mg/kg.

### Effect of tHGA on serum TNF-α level in LPS-induced BALB/c mice

**Figure 2.**
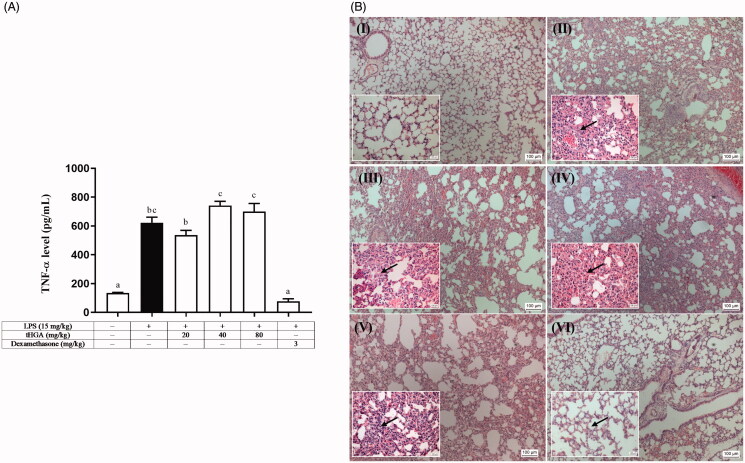
Effect of tHGA on (A) TNF-α level and (B) lung histopathological changes in LPS-induced BALB/c mice. Mice were pre-treated with different doses of tHGA or dexamethasone prior to LPS induction. After 6 h, the serum was collected to measure the level of TNF-α by using ELISA kit. Data are expressed in mean ± S.E.M. (*n* = 6), with groups that have no superscript letter in common are significantly different from each other (*p* ≤ 0.05). Representative H&E staining of lung sections of mice pre-treated with different doses of tHGA or dexamethasone under light microscope with 100× and 400× (small boxes). (I) Normal, (II) LPS control, (III) LPS + 20 mg/kg tHGA, (IV) LPS + 40 mg/kg tHGA, (V) LPS + 80 mg/kg tHGA and (VI) LPS + 3 mg/kg dexamethasone. Arrows indicate the infiltration of inflammatory cells.

Proinflammatory cytokines, such as TNF-α, play important roles in the inflammatory process. To test the effect of tHGA on inflammation, ELISA was performed. This test examines the secretion level of TNF-α in LPS-induced mice. Since the 2 mg/kg tHGA treatment did not significantly or consistently inhibit vascular leakage and leukocyte infiltration in the previous experiments, the doses for this assay were modified. Significant results were observed consistently at 20 and 100 mg/kg dosage, so pre-treatment at 20, 40 and 80 mg/kg was chosen for this assay. The ELISA analysis showed that LPS alone caused the mice to release more TNF-α. tHGA pre-treatment at all doses did not inhibit TNF-α secretion (Figure 2(A)). However, mice pre-treated with 3 mg/kg dexamethasone had significantly reduced TNF-α secretion. These findings demonstrate that all doses of tHGA failed to inhibit the release of the proinflammatory cytokine, TNF-α, in LPS-induced mice.

### Effect of tHGA on lung histopathological changes in LPS-induced BALB/c mice

The lungs of the experimental mice were harvested to assess the severity of organ inflammation upon tHGA pre-treatment and LPS induction via H&E staining. Under normal conditions, the mice displayed no abnormal histopathological change (Figure 2(B,I)). Upon LPS induction, the lung tissues showed marked inflammatory cell infiltration, a thickening of the alveolar wall, and lung tissue destruction (Figure 2(B,II)). tHGA pre-treatment at all tested doses were found ineffective at suppressing the histopathological changes induced by LPS (Figure 2(B,III–V)). On the other hand, dexamethasone pre-treatment was able to counteract the deleterious effect of LPS by maintaining a thin alveolar wall, resulting in fewer influxed inflammatory cells (Figure 2(B,VI)). Therefore, these findings indicate that tHGA was unable to inhibit organ inflammation in LPS-induced mice.

### Effect of tHGA on the survivability of LPS-induced BALB/c mice

**Figure 3.**
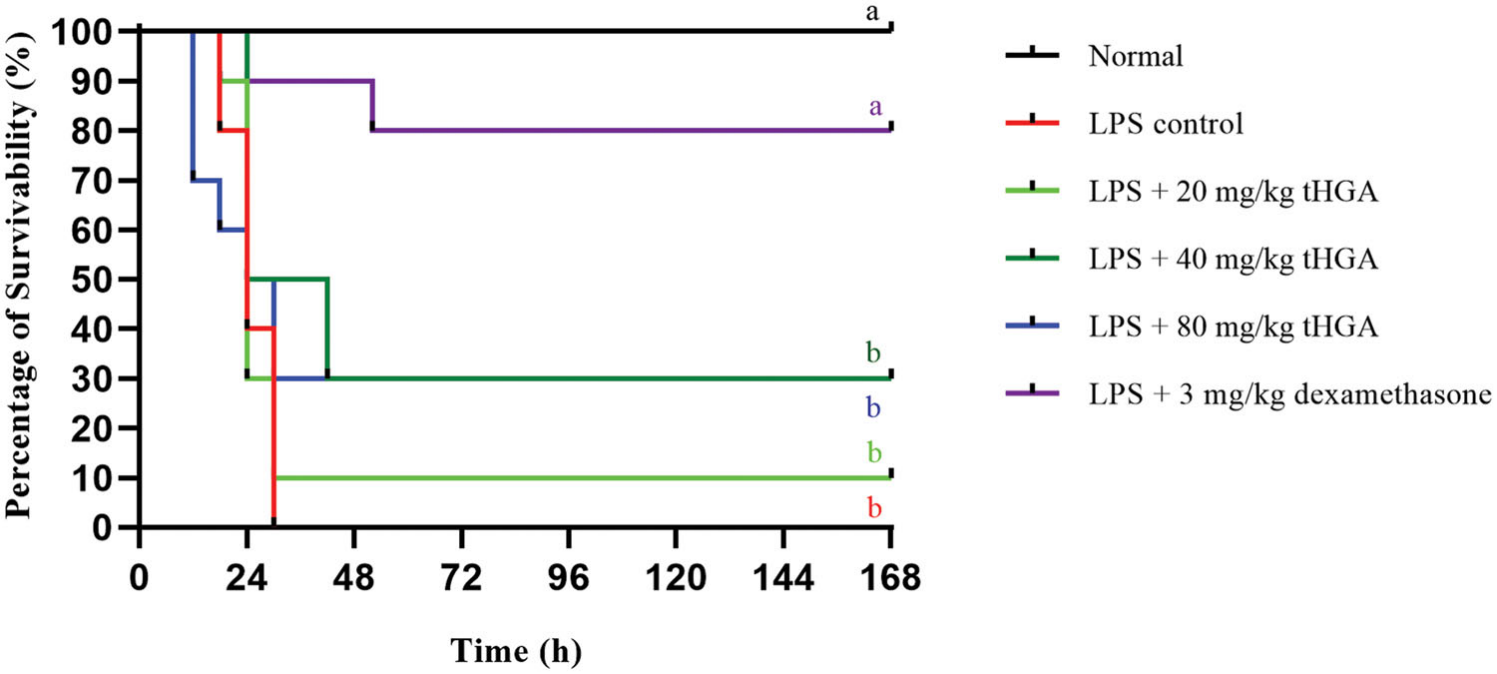
Effect of tHGA on survivability of LPS-induced BALB/c mice. Mice were pre-treated with different doses of tHGA or dexamethasone for 1 h prior to LPS induction. The mice were then returned to their individual cages and monitored for survivability at intervals of 6 h up to 168 h. Data are expressed in mean ± S.E.M. (*n* = 10), with groups that have no superscript letter in common are significantly different from each other (*p* ≤ 0.05).

A mouse model of endotoxemia was established to assess the effect of tHGA on LPS-induced lethality. The mice were pre-treated with different doses of tHGA after LPS induction, and their survivability was monitored every 6 h up to 168 h. As shown in Figure 3, all LPS-induced mice without treatment died from endotoxemia. However, upon pre-treatment with 20 mg/kg tHGA, 10% of the mice survived under LPS induction. The endotoxemic mice pre-treated with 40 and 80 mg/kg tHGA showed a survivability rate of 30%. However, this increment in survivability rate was not significant for all groups pre-treated with tHGA ranging from 20 to 80 mg/kg. Dexamethasone, which served as the drug control, significantly reduced the LPS lethality to the mice by 80%. These results suggest that the tHGA pre-treatment at all doses was unable to significantly prevent the endotoxemic mice from dying.

## Discussion

The endothelium is a monolayer of endothelial cells lining the inner vascular wall of blood vessels and the lymphatic system, so it is in direct contact with the blood or lymph, and circulating cells. Under normal physiological conditions, the endothelium works as a functional barrier that maintains homeostasis at an optimum level, including the regulation of inflammation, blood fluidity, platelet aggregation, coagulation and vascular tone (Daiber et al. 2017). Of these functions, endothelium’s role in regulating vascular inflammation has been the most extensively studied, as hyperinflammatory responses are deleterious to the hosts since it ultimately induces sepsis and tissue damage (Nedeva et al. 2019). LPS, a component found in the outer membrane of Gram-negative bacteria, is seen as a reason for the problem, as it can trigger the exacerbation of vascular inflammation and thus lead to the activation of endothelial cells through compromised adhesive properties and increased endothelial permeability (Li et al. 2016). Upon activation, the overproduction of proinflammatory mediators, such as leukotrienes, prostaglandins, cytokines and chemokines, leads to endothelial hyperpermeability; and thus, resulting in the unrestricted passage of plasma proteins (Abdulkhaleq et al. 2018). These events cause excessive fluid leakage that ultimately leads to endotoxemia and septic shock (Kottke and Walters 2016).

Vascular leakage is the primary hallmark of endotoxemia that eventually leads to sepsis. As tHGA was found to exhibit barrier protective effects *in vitro*, it is, therefore, natural to investigate such effects *in vivo*. Permeability is a pre-event that enables the movement of leukocytes across the vascular barrier from the vessel to the site of injury. Therefore, the effect of tHGA on vascular leakage in LPS-induced endotoxemic mice was studied further using Evans blue permeability assay. The results show that tHGA at all tested doses were effective at suppressing Evans blue permeation into the peritoneal cavity of LPS-induced mice. These results are consistent with our previous *in vitro* findings, thus supporting the ability of tHGA to preserve the vascular barrier integrity in endothelial cells (Chong et al. 2016).

Leukocyte adhesion to the endothelium is a pre-requisite for leukocyte infiltration or extravasation (Park et al. 2014; Su et al. 2017). tHGA has been proven to reduce monocyte adhesion and transmigration through the inhibition of CAM expression and prostaglandin production *in vitro* (Chong et al. 2016). To confirm this effect *in vivo*, the effect of tHGA on LPS-induced leukocyte infiltration was examined. The results reveal that tHGA at doses of 20 and 100 mg/kg had a profound effect in the suppression of leukocyte influx into the peritoneal cavity of LPS-induced mice. These findings agree with our previous investigation on vascular leakage, where tHGA was observed effective at suppressing the permeation of Evans blue across the LPS-induced vascular barrier. Leukocytes can either circumvent the vascular barrier by passing through the gaps between the destabilized cell–cell junctional proteins via paracellular route, or directly transcytosing through the body of the endothelial cells via transcellular route. In other words, leukocyte infiltration can occur in the presence or absence of vascular leakage, mediated by distinct molecular pathways. However, paracellular route involving junctional proteins has been demonstrated to be the prevalent way of leukocytes infiltrating across various inflamed tissue into the bloodstream (Vestweber 2012; Vestweber et al. 2014). Therefore, based on the consistent findings of Evans blue permeability and leukocyte transmigration assays, it is deduced that leukocyte infiltration in this experimental model is most likely occur through paracellular route during the manifestation of vascular leakage. Collectively, these findings are in agreement with our earlier *in vitro* study, thus endorsing the effectiveness of tHGA in attenuating leukocyte transmigration across the vascular barrier based on the preservation of endothelial integrity in the *in vitro* model of LPS-induced endothelial cells (Chong et al. 2016).

As tHGA was observed to be significantly effective at suppressing vascular leakage and leukocyte infiltration at 20 and 100 mg/kg, this result prompted the researchers to test the effect of tHGA doses that fall within this range. Hence, tHGA pre-treatment at 20, 40 and 80 mg/kg were used in subsequent experimental assays, including ELISA, the histopathological analysis and the survivability assay. Earlier investigations have shown that tHGA could preserve vascular permeability, which in turn, suppressed leukocyte infiltration across the vascular wall of endotoxemic mice. As excessive fluid leakage is an important hallmark in sepsis, this study hypothesized that tHGA, due to its effects on vascular inflammation and endothelial permeability, could be considered a potential therapeutic agent for controlling sepsis. To test this hypothesis, this study assessed the effects of tHGA on several inflammatory parameters, including the secretion of proinflammatory TNF-α and lung histopathological changes. In the presence of stimuli such as LPS, the levels of proinflammatory cytokines, including TNF-α, IL-6 and IL-10 are elevated because activated macrophages start producing them more (Chae 2018; Wu et al. 2018). These inflammatory mediators play a dominant mediating role in acute inflammatory responses and sepsis. Therefore, drugs that can regulate or alter the production of inflammatory mediators can be considered potential candidates for the prevention or mitigation of septic shock. Among the proinflammatory cytokines, TNF-α has been identified as a particular mediator of sepsis due to its pathogenesis in LPS-induced septic shock (He et al. 2020). To test this hypothesis, the effect of tHGA on serum TNF-α level in endotoxemic mice was further assessed via ELISA. The results show that LPS administration induced an acute inflammatory response, accompanied by the elevation of TNF-α secretion. Although the optimal production of TNF-α aids the innate immune response, overproduction results in acute phase endotoxemia and tissue injury, septic shock and even death (Liu et al. 2019). Unfortunately, tHGA pre-treatment at all doses failed to suppress the production of TNF-α in LPS-induced mice. As TNF-α mediates the production of nitric oxide, this finding is somewhat in line with a previous study from Abas et al. (2010) which reported that tHGA did not exert significant inhibitory effect on the production of nitric oxide from LPS-stimulated RAW264.7 cells.

Studies have extensively identified the crucial role of LPS-provoked progressive secretion of proinflammatory mediators, such as TNF-α, in multiple organ dysfunction and lethality during endotoxemia (Kumari et al. 2017). Inhibition of TNF-α overproduction was proven to mitigate multiple organ failure and improve the survival of mice with endotoxemia (Penalva et al. 2017). Of the organs affected, the lungs are the most susceptible to failure. The prominent features of LPS-induced lung injury include neutrophil infiltration and oedema (Lingaraju et al. 2015). LPS induction results in the marked thickening of the alveolar wall, attributed to the influx of inflammatory cells (Huang et al. 2015). However, tHGA pre-treatment at all tested doses failed to improve the morphological changes induced by LPS. Therefore, tHGA does not possess protective effect against LPS-induced organ dysfunction. Collectively, these outcomes are likely attributed to the incapability of tHGA in attenuating TNF-α elevation in LPS-induced mice.

The current findings show that tHGA managed to significantly suppress vascular leakage and leukocyte infiltration across the vascular barrier in LPS-induced mice. However, tHGA failed to inhibit the secretion of proinflammatory TNF-α and organ dysfunction upon LPS induction. These findings are somewhat in line with our previous *in vitro* studies. That is, tHGA exerts barrier protective effects primarily due to its direct action on the endothelial barrier. However, tHGA does not exert any effect on proinflammatory cytokines and chemokines, such as the monocyte chemoattractant protein-1 (MCP-1) (Chong et al. 2016). Besides, our on-going study demonstrated that tHGA preserved the expression of junctional proteins such as zonula occluden-1 (ZO-1), occludin and vascular endothelial-cadherin (VE-cadherin) in LPS-induced endothelial hyperpermeability, via the inhibition of activation of NF-κB, p38 MAPK and myosin light chain (MLC) pathways. Therefore, this result warrants a subsequent study on tHGA in an endotoxemia model comprising all the aforementioned components. To test this hypothesis, the effect of tHGA on LPS-induced lethality was examined using endotoxemic mice. This experimental approach was selected because putative therapeutic modulators of the sepsis syndrome are frequently evaluated first in models involving LPS induction (Gatica-Andrades et al. 2017; Lan et al. 2017). In this study, tHGA pre-treatment at all selected doses failed to significantly rescue the experimental mice in the LPS-induced endotoxemia model from death. These findings are not surprising, as endotoxemia is a condition that involves a complex interplay of vascular and endothelial dysfunction, sub-clinical end-organ dysfunctions, and inflammatory protein and cytokine dysregulation (Rodriguez-Rosales et al. 2019; van Lier et al. 2019). The findings show that tHGA could only inhibit vascular leakage and leukocyte infiltration and was unable to suppress TNF-α overproduction or the histopathological changes in the lungs. Therefore, the mode of action of tHGA on endotoxemia is limited to the attenuation of LPS-induced vascular and endothelial barrier disruption but is not workable on major inflammatory cytokines, such as TNF-α, which leads to systemic inflammation (He et al. 2020). These findings collectively and indirectly prove that the failure of tHGA in rescuing endotoxemic mice from lethality might be due to its incapability to suppress the overproduction of TNF-α; and thus, leading to a failure in protecting organ dysfunction upon LPS induction. This effect is in stark contrast to dexamethasone, which significantly improved the survivability of the endotoxemic mice. This result is in agreement with that of Cui et al. (2015), who demonstrated the ability of dexamethasone to inhibit the activation of proteins that mediate sepsis-induced vascular hyperpermeability *in vivo*. Thus, it is not surprising that dexamethasone exhibited the same potent protective effect against LPS-induced endotoxemia in this study.

Nonetheless, there are several limitations in this study. First, only lungs were assessed for histopathological changes because the lungs are the most susceptible organs to multiple organ failure among all the organs affected. This is attributed to the rich capillary bed in the lungs that have an interface proximate to the alveolar epithelial layer (Herrero et al. 2017). Also, tHGA has been demonstrated to be effective in attenuating allergic airway inflammation (Ismail et al. 2012, 2019) as well as airway remodelling (Lee et al. 2017) in ovalbumin-sensitized mice. Sim et al. (2018) also reported that tHGA was capable of attenuating TNF-α-induced pulmonary epithelial barrier dysfunction which is an important hallmark in the pathogenesis of acute lung injury (ALI) and acute respiratory distress syndrome (ARDS). Therefore, inspection of the lungs could easily provide an indication of the protective effects of tHGA against LPS-induced organ dysfunctions. Second, only TNF-α production was examined as TNF-α is the major inflammatory cytokine which leads to systemic inflammation (He et al. 2020). It is recommended that future studies should further examine the mode of action of tHGA in reducing LPS-induced vascular leakage and leukocyte infiltration, including examination of the effects of tHGA on the rolling, adhesion and migration of leukocytes in the cremaster muscle postcapillary venules using intravital microscopy.

## Conclusions

The inhibition of vascular leakage and exacerbated inflammatory responses are considered promising molecular targets for the treatment of various vascular inflammatory diseases. Herein, tHGA has been proven effective at preserving vascular permeability and suppressing leukocyte infiltration across the vascular wall of LPS-induced endotoxemic mice. However, tHGA was unable to rescue the endotoxemic mice from lethality due to its incapability to inhibit the elevated production of TNF-α, as well as its failure to prevent organ dysfunction, in LPS-induced endotoxemic mice. Therefore, tHGA should be developed further before it can be used as a potential therapeutic agent for diseases related to prolonged or uncontrolled vascular leakage. Until then, it is recommended that tHGA be combined with anti-inflammatory agents to treat vascular inflammatory disorders, such as endotoxemia and sepsis.
